# Metallopeptidases of *Toxoplasma gondii*: *in silico* identification and gene expression

**DOI:** 10.1051/parasite/2018025

**Published:** 2018-05-08

**Authors:** Sandie Escotte-Binet, Antoine Huguenin, Dominique Aubert, Anne-Pascaline Martin, Matthieu Kaltenbach, Isabelle Florent, Isabelle Villena

**Affiliations:** 1 EA 7510, ESCAPE, Laboratory of Parasitology-Mycology, University of Reims Champagne-Ardenne, 51100 Reims France; 2 Laboratory of Parasitology-Mycology, Toxoplasmosis National Reference Center, Toxoplasma Biological Resource Center, Maison Blanche Hospital, 51100 Reims France; 3 UMR7245 CNRS-MNHN, National Museum of Natural History, Department Adaptations of the Living, 75005 Paris France

**Keywords:** *Toxoplasma gondii*, metallopeptidase, endopeptidase, carboxypeptidase, aminopeptidase, enzymatic activity

## Abstract

Metallopeptidases are a family of proteins with domains that remain highly conserved throughout evolution. These hydrolases require divalent metal cation(s) to activate the water molecule in order to carry out their catalytic action on peptide bonds by nucleophilic attack. Metallopeptidases from parasitic protozoa, including *Toxoplasma*, are investigated because of their crucial role in parasite biology. In the present study, we screened the *T. gondii* database using PFAM motifs specific for metallopeptidases in association with the MEROPS peptidase Database (release 10.0). In all, 49 genes encoding proteins with metallopeptidase signatures were identified in the *Toxoplasma* genome. An Interpro Search enabled us to uncover their domain/motif organization, and orthologs with the highest similarity by BLAST were used for annotation. These 49 *Toxoplasma* metallopeptidases clustered into 15 families described in the MEROPS database. Experimental expression analysis of their genes in the tachyzoite stage revealed transcription for all genes studied. Further research on the role of these peptidases should increase our knowledge of basic *Toxoplasma* biology and provide opportunities to identify novel therapeutic targets. This type of study would also open a path towards the comparative biology of apicomplexans.

## Abstract (fr)

Les métallopeptidases sont une famille de protéines dont les domaines restent hautement conservés tout au long de l’évolution. Ces hydrolases nécessitent un ou plusieurs cations métalliques divalents pour activer la molécule d’eau afin de réaliser leur action catalytique sur la liaison peptidique par une attaque nucléophile. Les métallopeptidases provenant de protozoaires parasites, y compris *Toxoplasma*, sont étudiées en raison de leur rôle crucial dans la biologie du parasite. Dans la présente étude, nous avons examiné la base de données de *T. gondii* en utilisant des motifs PFAM spécifiques des métallopeptidases en association avec la base de données de peptidases MEROPS (version 10.0). Quarante-neuf gènes encodant des protéines avec des signatures de métallopeptidases ont été identifiés dans le génome de *Toxoplasma*. L’utilisation d’Interpro Search a permis de découvrir l’organisation des domaines / motifs ainsi que les orthologues présentant la plus haute similarité par BLAST et ont été utilisés pour l’annotation. Ces 49 métallopeptidases de *Toxoplasma* ont été regroupées dans les 15 familles décrites dans la base de données MEROPS. L’analyse expérimentale de l’expression de leurs gènes au stade tachyzoïte a révélé la transcription de tous les gènes étudiés. D’autres recherches sur l’implication de ces peptidases devraient accroitre notre connaissance de la biologie fondamentale de *Toxoplasma* et fournir des opportunités pour identifier de nouvelles cibles thérapeutiques. Une telle étude ouvre également la voie à la biologie comparée des Apicomplexa.

## Introduction

*Toxoplasma gondii* is an obligate intracellular apicomplexan protozoan parasite that is responsible for toxoplasmosis in humans and animals. Although toxoplasmosis is generally clinically asymptomatic in healthy individuals, it may cause severe complications and become opportunistic in immunocompromized hosts, such as AIDS and transplant patients. It can also cause severe congenital infections. Proteases, including metallopeptidases, play major roles in all organisms, catalyzing a broad spectrum of important biological reactions, including protein metabolism, immune reactions, and tissue remodeling for example. It is not surprising, therefore, that proteases have been found in species from viruses to humans. In parasites, besides basic roles in eukaryotic cell biology and physiology, proteases fulfill specific functions linked to the parasitic way of life, facilitating invasion of host tissues or parasite egress, allowing parasites to digest host proteins, helping parasites to evade the host immune response, and preventing blood coagulation among others [16,37,45–46]. As seen with other apicomplexan parasites such as *Plasmodium, Eimeria* and *Cryptosporidium*, toxoplasmic proteases could thus be considered potential therapeutic targets in light of this involvement in host-parasites interactions [16,37,45–46]. Metallopeptidases represent a very diverse catalytic type of peptidase and are classified in the MEROPS database (https://www.ebi.ac.uk/merops/) based on homologous sets of peptidases containing related sequences that are grouped together in families, which are then grouped in clans based on their related primary structures [56]. All known metallopeptidases have been divided into 16 different clans as described in MEROPS: MA (divided in MA(E) also called gluzincins and MA(M) called metzincins sub-clans), MC, MD, ME, MF, MG, MH, MJ, MM (with a motif like that of clan MA but bound to plasma membranes), MN, MO, MP, MQ, MS, MT, and M- which includes metallopeptidases which are not yet well characterized. Only a few of these clans are represented in *T. gondii*. Studies on *T. gondii* metallopeptidases remain scarce today, whereas complete surveys of protease homologs in *Plasmodium falciparum* [16,66] and *Eimeria tenella* predicted proteomes [31] have been published. To date, seven metallopeptidases have been experimentally explored in *T. gondii* [5,23,25,29–30,34,67,68–69]. The ToxoDB database (http://toxodb.org/toxo/, Release 29), that gathers *T. gondii* genome and post-genome data for numerous strains, provides an invaluable resource to investigate the most complete set of metallopeptidases for this parasite. Using human and protozoan metallopeptidase sequences and peptidase family domains (PFAM motifs [19]) defined in the MEROPS database, we identified, in ToxoDB, 49 putative toxoplasmic metallopeptidases clustered into 15 families corresponding to 7 clans. Expression of these metallopeptidase genes in the tachyzoite stage was then evaluated by PCR and RT-PCR assays.

## Materials and methods

### T. gondii *metallopeptidase identification,* in silico *analysis and classification*

In this manuscript, we chose to classify metallopeptidases according to their MEROPS classification in families, beyond their amino-, carboxy- or endopeptidase predicted activity. Putative metallopeptidase *Toxoplasma* genes were identified from the *T. gondii* ToxoDB database, (http://toxodb.org/toxo/, Release 29) using peptidase family domain (PFAM motifs) recorded in the MEROPS Database (https://www.ebi.ac.uk/merops/) for this family of enzymes. The TGME49 genome was chosen as a reference in our study. The domain/motif organization of predicted proteases was studied using the Interpro Search (http://www.ebi.ac.uk/interpro/). At the end of this search, a total of 49 genes encoding proteins with metallopeptidase signature motifs were identified in the *Toxoplasma gondii* ME49 genome. They were subsequently assigned to families and sub-families of metallopeptidase annotations by amino acid sequence comparisons using the BLASTp program in the Washington University (http://www.ebi.ac.uk/Tools/blast) and the BLAST MEROPS server using the MEROPS Database.

The deduced amino acid sequences of these putative *Toxoplasma* metallopeptidases proteins were aligned with sequences from other organisms according to the ClustalW multiple sequence alignment algorithm on the EMBL-EBI website (European Bioinformatics Institute, www.ebi.ac.uk/Tools/clustalw2) using the Blosum62 matrix. The prediction of protein localization sites in parasites was performed by using a computer program Psort II (http://www.psort.org/).

### Parasites

The RH *T. gondii* strain (genotype I) was used throughout our experiments. Tachyzoites were obtained by inoculation of *T. gondii* in the intraperitoneal cavity of female Swiss mice. The animal housing facility is accredited according to French regulations (approval No. B 51-454-4). The experimental protocol for inoculation was approved by the local Ethics Committee for Animal Experiments (CEEA RCA No. 56) and is referenced under state law under protocol number 56-2012 -16.

### Design of specific primers for each metallopeptidase sequence

Primers were designed based on the selective sequences of the RH *T. gondii* genomic DNA (gDNA). Positions of introns in putative metallopeptidase genes were obtained by alignment of gDNA with complementary DNA (cDNA). One pair of primers was designed per gene following two conditions if possible: the pair of primers should flank a genomic region spanning an intron and/or amplify the metallopeptidase catalytic domain. All primer pairs were designed using Primer Pro 3.4 software (www.changbioscience.com/primo/primo.html). The primers used to assess metallopeptidase gene expression are listed in Table 1 with their corresponding gene.

**Table 1:**
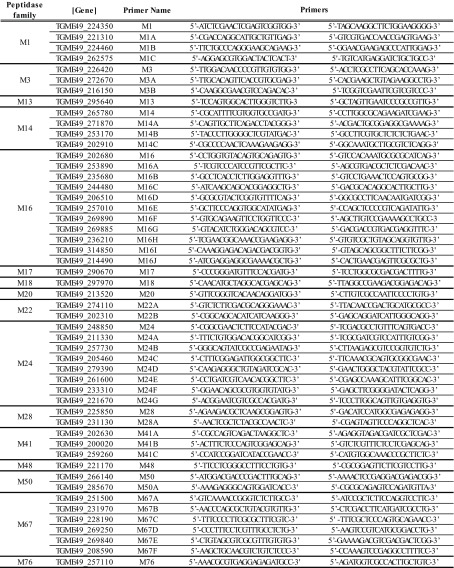
Metallopeptidase primers used for the PCR and RT-PCR. Gene: gene nomenclature in ToxoDB release 29.

### Polymerase chain reaction (PCR) of metallopeptidases sequences

Genomic DNA was extracted from purified RH *T. gondii* tachyzoites using the QIAamp^®^ DNA Mini Kit (Qiagen, Courtaboeuf, France), following the manufacturer’s instructions. Amplifications were performed using 1 μL of cDNA or 3 μL of gDNA (10 ng/μL), 50 pmol of each primer and 2U of Taq DNA polymerase (Invitrogen^TM^ Life Technologies) in 50 μL PCR reaction containing 1×PCR Buffer (20 mM Tris–HCl (pH 8.4), 50 mM KCl, 1.5 mM MgCl_2_), and 200 μM of dATP, dTTP, dGTP, dCTP. The template was subjected to 35 cycles (94 °C for 30 s, 60 °C for 45 s and 72 °C for 2 min) followed by a final 10 min extension at 72 °C. PCR products were analyzed by electrophoresis in 1× TBE buffer on 2% agarose gel stained with 0.5 μg/mL ethidium bromide and photographed under ultraviolet light (Phospho imager, Biorad). SAG1 tachyzoite transcript was used as an RT-PCR positive control (SAG1S 5′-caatgtgcacctgtaggaagc-3′, SAG1R 5′-tgggcaggtgacaacttgatt-3′). A negative control containing all reagents, except cDNA, was also included. The presence of gDNA contamination in cDNA samples was verified by PCR using primer pairs which amplify intron(s)-containing regions.

### Reverse transcription-PCR

Total RNA was isolated from tachyzoites using the RNeasy^®^ Mini Kit (Qiagen). Prior to reverse transcriptase (RT)-PCR analysis, total RNA was treated at room temperature for 15 min with RNase-free DNase I (Invitrogen^TM^ Life Technologies). Total RNA samples of 1 μg, denatured at 65 °C for 10 min, were reverse transcribed at 42 °C for 50 min in a total volume of 20 μL using oligo-(dT)18 as the primer with 200U Superscript^TM^ II reverse transcriptase (Invitrogen^TM^ Life Technologies). Following heat inactivation at 70 °C for 15 min, the reverse transcribed mRNA (cDNA) mixture was incubated with 2U of *Escherichia coli* RNase H at 37 °C for 20 min to remove complementary RNA to the cDNA. A negative control containing all reagents, except total RNA, was also included for each experiment.

## Results and discussion

**Figure 1:**
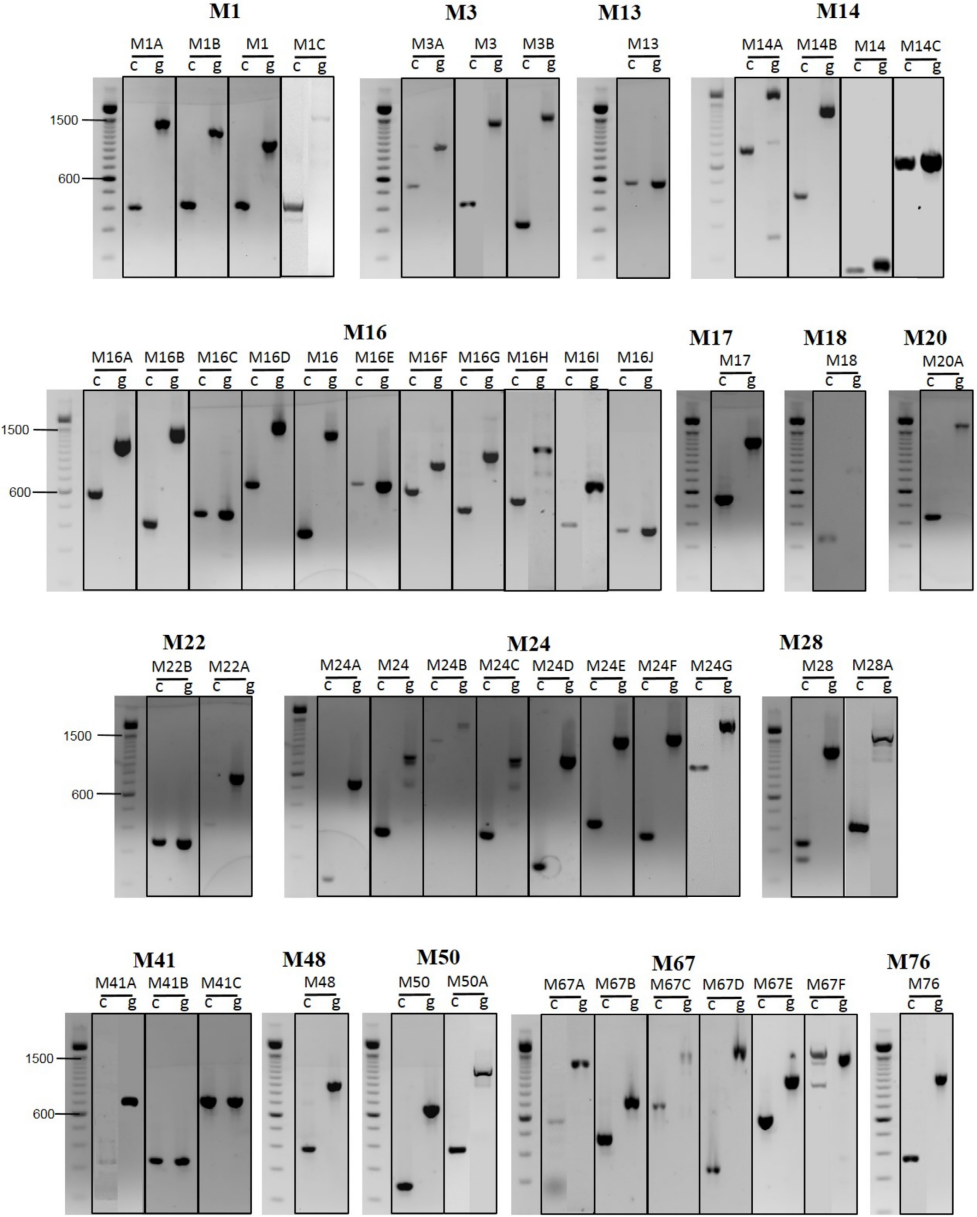
Metallopeptidase gene expression in extracellular toxoplasmic tachyzoites by RT-PCR. Products of the expected size were observed for all primers, using either cDNA and gDNA as templates. As a further control for the presence of contaminating gDNA, primers of each gene were designed to amplify fragments of distinct length from cDNA(c) and gDNA(g) due to the presence of introns. Molecular size standards are indicated to the left.

The *T. gondii* reference genome database ToxoDB was screened to identify putative metallopeptidase sequences. In all, 49 metallopeptidases containing PFAM domains that characterize the metallopeptidase enzyme superfamily were identified. The genome of the RH strain (genotype I) shares high similarity to the archived genomes of the *T. gondii* GT1 (genotype I), ME49 (genotype II) and VEG strains (genotype III) [35]. We then decided to investigate metalloprotease expression in total RNA from RH tachyzoites by conventional RT-PCR. To do so, gene-specific primer pairs flanking a region spanning intron(s) were designed in order to amplify fragments of distinct length from cDNA and gDNA templates. Amplifications with these different primers pairs yielded PCR and RT-PCR products of expected sizes for each gene (Figure 1). These results confirmed their transcription and are in agreement with the currently proposed intron-exons gene model boundaries in ToxoDB. In view of the high structural diversity seen in metallopeptidase families, putative metallopeptidases from the *T. gondii* genome database were assigned according to the MEROPS classification, as described below.

In this study, we used the MEROPS Nomenclature system (release 10.0) as described in Rawlings et al. (2016) [56]. In this system, proteases are classified into 8 catalytic superfamilies (aspartic, cysteine, glutamic, metallo-, mixed catalytic type, serine, threonine, and unknown catalytic type peptidases), and metallopeptidases into 16 clans based on their related structures. Metallopeptidases from the *T. gondii* genome database (ToxoDB) were therefore classified based on their domain organization and sequence similarities to metallopeptidases from other organisms. We have found 49 putative metallopeptidases as shown in Table 2 (see also Appendix 1), which details protease MEROPS clans and families, number of metal ions, *T. gondii* ME49 gene ID, chromosomal location, protein length (amino acids), ToxoDB product description, protease homolog with highest BLAST score using BLASTp program combined with MEROPS BLAST server, primer name correspondence, alias, related publication and PFAM ID, and signal peptide presence/prediction. A total of 49 putative metallopeptidases have thus been identified and ascribed to 15 metallopeptidases families described in the MEROPS database: four M1, three M3, one M13, four M14, eleven M16 (the most represented family), one M17, one M18, one M20, eight M24, two M28, three M41, one M48, two M50, six M67 and one M76 peptidase families.

**Table 2:**
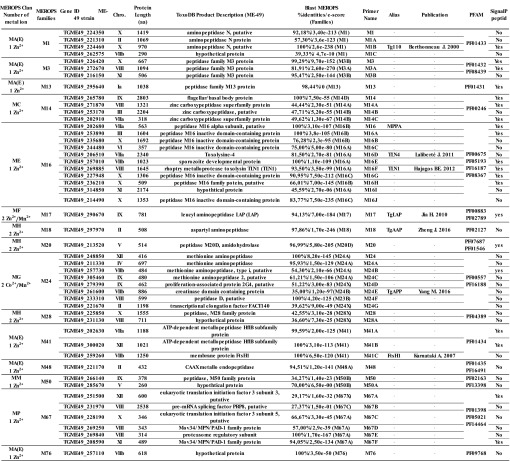
Metallopeptidase genes identified and classified in the *T .gondii* genome database (strain ME-49, genotype II). We used Pfam motifs (http://pfam.xfam.org/) in association with the MEROPS Database to screen the *T. gondii* database (http://toxodb.org/toxo/, Release 29). The motif organization of predicted peptidases was studied using the InterProScan Search (http://www.ebi.ac.uk/interpro/) and family assignment is based on MEROPS − the peptidase Database − classification (https://www.ebi.ac.uk/merops/).

Hereafter, we describe our results concerning each of these 15 families found in *T. gondii* genome.

### M1 Peptidase family (Aminopeptidase N family)

M1 family peptidases, also called membrane alanyl aminopeptidase (aminopeptidase N), are dependent on a single zinc ion for activity, and catalyze amino acid cleavage from amino-termini of protein or polypeptide substrates. These aminopeptidases are involved in several biochemical processes, including protein maturation and activation. This M1 family of metallopeptidase enzymes (clan MA(E)) presents 2 key signatures: H**E**xxH(x)_18_E, the active site motif in which the 2 histidines and the last glutamic acid (underlined) bind zinc atom and the first glutamic acid (bold) is involved in catalysis, and an upstream GAMEN motif involved in substrate recognition.

**Figure 2:**
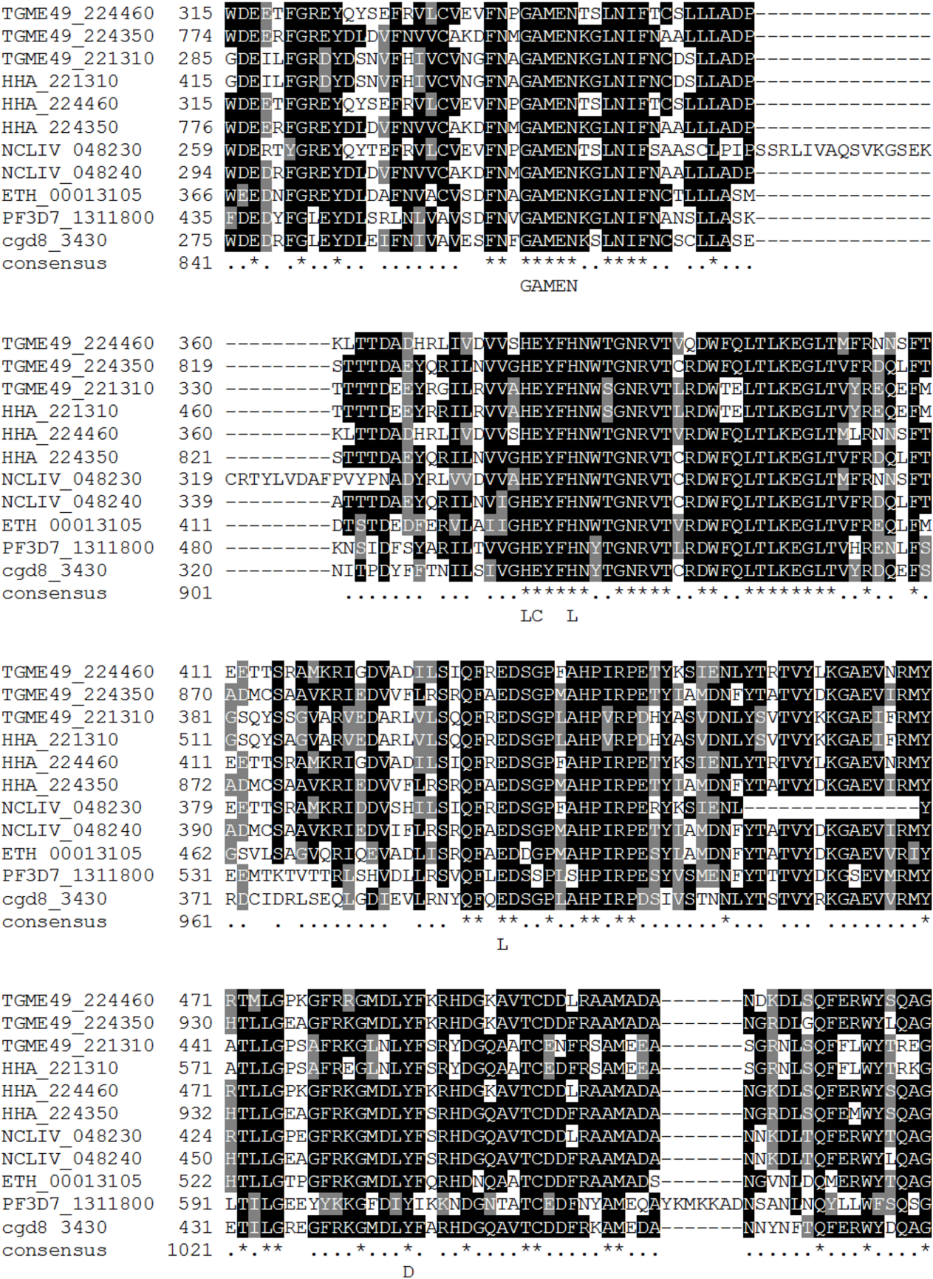
Multiple sequences alignment from *T. gondii* aminopeptidase N (M1 peptidase family) and several selected members of the M1 family of zinc-metallopeptidases: *P. falciparum* (PF3D7_1311800 and PF3D7_1472400), *T. gondii* (TGME49_221310, TGME49_224350, and TGME49_224460), *N caninum* (NCLIV_048240 and NCLIV_048230), and *C. parvum* (cgd8_3430). Amino acid positions identical between these sequences and the *T. gondii* sequence are in darkened letters. Identical (black background) and conserved (grey background) amino acids between all sequences are indicated. The position of the conserved putative zinc ion ligands (L), the conserved glutamyl residue required for catalytic activity (C), and the conserved putative proton donor (D) are indicated in bold on the bottom line. The amino acid numbers for each sequence are indicated on the left. The position of gaps is indicated by full colons. Alignments were performed using the ClustalW2 algorithm (www.ebi.ac.uk/Tools/clustalw2) with the Blosum 62 matrix.

Four *T*. *gondii* peptidases from ToxoDB can be ascribed to this M1 peptidase family (TGME49_221310 (*Tg110*), TGME49_224460, TGME49_224350, and TGME49_262575) but only three display both typical HExxH(x)_18_E and GAMEN signatures (Figure 2). TGME49_262575 is highly atypical and very small (290 amino acid). It may be incomplete in ToxoDB but is, however, conserved among coccidia which is intriguing and deserves further investigation. Interproscan analysis indicates a leukotriene A4-hydrolase domain (Superfamily domain SSF63737), classified within the M1 peptidase family. A *T. gondii* aminopeptidase, named *Tg110*, and able to cleave L-Arg-AMC, L-Leu-AMC, and L-Tyr-AMC (aminopeptidase substrates) was described experimentally by Berthonneau in 2000 [5]. *Tg110* was identified in cell-free extracts and was purified using high-performance liquid chromatography. Its optimal activity was at pH 7.4 and it was strongly inhibited by classical metallopeptidase inhibitors (EDTA and o-phenanthroline). The purified enzyme exhibited a pI of 4.7 and had an apparent molecular weight of 110 kDa. These features are in agreement with theoretical values for TGME49_224460 (Table 2, see also Appendix 1). Interestingly, *Tg110* was detected in human sera from patients undergoing toxoplasmosis, suggesting involvement in infection response [5]. As of today, no function has been ascribed for any M1 peptidase family from *T. gondii*.

The importance of M1 family aminopeptidases has been recognized in closely related protozoan species including PF3D7_1311800 (PfA-M1) from *P. falciparum* [1,3,7,14–15,20,24,43,62], NCLIV_048240 (NcAPN1) and NCLIV_048230 (NcAPN3) from *N. caninum* [22], cgd8_3430 from *C. parvum* (strain Iowa II) [49], and ETH_00013105 and ETH_00013105 from *E. tenella* (strain Houghton) also called EtAPN1 [22] and EtAPN2 [31]. The role of PfA-M1 is largely documented. PfA-M1 is found in various locations in the malaria parasite, such as the cytoplasm, food vacuole, parasitophorous vacuole and nucleus [1,3,14,43]. This M1 aminopeptidase has been mainly involved in parasite metabolism in the last steps of hemoglobin degradation [54] but also in parasite development [48]. EtAPN1 is an active protease during *Eimeria* parasite sporulation [18]. Using bestatin, a well-known broad-spectrum inhibitor of metalloaminopeptidases, on *E. tenella* infected culture *in vitro*, a strong inhibition of parasite development but not of the invasion process was observed [22].

### M3 Peptidase family (thimet oligopeptidase and oligopeptidase F families)

M3 peptidases, also belonging to the MA(E) clan, display a highly conserved signature FH**E**xGH(x)_2_H(x)_12_G(x)_5_D(x)_2_ExPS(x)_3_E, including the H**E**xxH motif, in which **E** (bold) is involved in catalysis and the two underlined H and a C-terminally located E residue act as zinc-binding ligands [56]. This type of endopeptidase only hydrolyzes oligopeptides that contain no more than 20 amino acid residues. The M3 peptidase family is involved in peptide degradation, bioactive neural-peptide synthesis, and cleavage of signal peptides. Most of the M3 peptidases are synthetized without signal peptides, except Mitochondrial Intermediate Peptidase (MIP) which possess a typical amino-terminal mitochondrial leader peptide recognized and cleaved by the mitochondrial processing peptidase. The main role of M3 peptidases is to cleave short peptidic substrates in the cytoplasm, whereas MIP resides in the mitochondrial intermembrane space and cleaves N-terminal octapeptides from proteins during their import into mitochondria. The M3 family is divided into three sub-families: M3A also called thimet oligopeptidases (including neurolysin and MIP), M3B called oligopeptidases F and M3C called Pz-peptidase A [56].

During *T. gondii* asexual development, an oligopeptidase F has been identified by the use of microarrays, and may be involved in the regulation of bradyzoite-specific metabolic pathways, as found in bacteria [13].

Three M3 family peptidases were found in ToxoDB (TGME49_272670, TGME49_226420 and TGME49_216150). By sequence homology, TGME49_272670 would belong to the M3A peptidase sub-family, whereas the two others could belong to the M3B oligoendopeptidase F subfamily (Table 2, see also Appendix 1). These enzymes are predicted to be localized in matrix mitochondria for TGME49_272670 and in the parasite cytoplasm for TGME49_226420 and TGME49_216150 (PSORT II prediction). As of today, no enzyme from the M3 peptidase family has been further experimentally described for any apicomplexan.

### M13 Peptidase family (Neprilysin family)

Also belonging to the MA(E) clan, the M13 family (also named neprilysin family) is a large group of zinc-metallopeptidases which present highly conserved sequences, including the HExxH motif and a C-terminally located E residue, in which the underlined amino-acids provide the three zinc ligands, and the catalytically important GENIAD and VNAFY motifs [6]. M13 peptidases are endopeptidases which are responsible for the inactivation and/or activation of peptide signaling events on cell surfaces. Current knowledge suggests that all peptidases in family M13 are restricted to acting on substrates of no more than about 40 residues [56]. These enzymes appear to be synthetized in active forms, without proenzyme forms. The majority of currently described M13 endopeptidases are type II integral transmembrane zinc-metallopeptidases. Homologs are known from all kingdoms of life, but principally so far from bacteria and animals.

As of today, no enzyme from the M13 peptidase family has been described in *T. gondii*, nor in other apicomplexa. In our study, one *T*. *gondii* M13 peptidase was found in ToxoDB (TGME49_295640) that is predicted to be localized in the mitochondrial matrix space according to PSORT II prediction.

### M14 Peptidase family (carboxypeptidase A1, carboxypeptidase E, gamma-D-glutamyl–meso-diaminopimelate peptidase I and cytosolic carboxypeptidase 6 families)

Clan MC contains metallocarboxypeptidases of the M14 family. Within the M14 family, sequence conservation around the zinc ligands and catalytic residues allowed to distinguish four sub-families: M14A, M14B, M14C, and M14D. Most of the carboxypeptidases are synthetized without signal peptides, but with N-terminal propeptides that must be processed to release active enzymes. These carboxypeptidases hydrolyze single C-terminal amino acids from polypeptide chains.

Currently, four *T*. *gondii* M14 family peptidases have been found in ToxoDB that are thus predicted to display carboxypeptidase functions. Among them, two are indeed characterized by an EC number. TGME49_253170 is characterized as EC 3.4.17.12 (carboxypeptidase M) that is predicted to cleave the amino acids arginine or lysine at the C-terminal of peptidic substrates. In contrast, the TGME49_202910 carboxypeptidase is characterized as EC 3.4.17.1 (carboxypeptidase A), which is predicted to cleave all the other amino acids located at the C-terminal of peptidic substrates except arginine, lysine and proline [56].

### M16 Peptidase family (pitrilysin, mitochondrial processing peptidase beta-subunit and eupitrilysin family)

Clan ME includes the M16 peptidase family in which two of the three zinc ligands are present in the motif Hxx**E**H. The complete M16 peptidase family catalytic site signature is Hxx**E**H_74_E in which the two underlined histidines and the last underlined glutamate are zinc binders and the first glutamate (bold) is involved in the catalytic reaction. This family consists of three sub-families named M16A, M16B, and M16C, in which the differences lie in the precise architecture of the catalytic sites. Members of the M16A and M16C families are composed of four domains in which only one possesses a zinc binding site. However, the members of the M16B family are heterodimers composed of two identical subunits each of which possesses a zinc binding site. Within the M16B family, MPP peptidases (Mitochondrial Processing Protease) are the most represented enzymes. As their name suggests, they are involved in proteolytic processing in mitochondria. They act with the IMP (Inner Membrane Peptidase) and MIP (Mitochondrial Intermediate Peptidase) to allow protein targeting in the different mitochondrial sub-compartments [21].

In this *in silico* study, 11 proteases were identified, characterized by the Hxx**E**H motif, also called “reverse catalytic signature”. Peptidases of the M16A family have been found in different parasites and particularly in *T.* *gondii*, where they are located in the rhoptries [9].

Two metallopeptidases have been described in *T.* *gondii* as belonging to the M16A family: toxolysin-1 (TGME49_269885) and toxolysin-4 (TGME49_206510). Toxolysin-1 is a zinc metalloprotease secreted from rhoptries [9]. It presents a pro-domain in its N-terminal region responsible for its targeting to this organelle. By constructing mutants of the gene encoding this protease, Hajagos et al. showed that this protease is not essential for parasite *in vitro* growth nor *in vivo* virulence [23]. Toxolysin-4, stored in micronemes, is released in response to an increase in Ca^2+^ level and could play a role during invasion [34]. This protease appears, in addition, to undergo a complex maturation process as six forms of this protease have been identified ranging from 260 kDa (precursor) to 34 kDa (degradation metabolite) [34].

No protease belonging to the M16B or M16C family has been described to date in *T.* *gondii*. Two M16C peptidases of *P. falciparum* are particularly well described: falcilysin (PF3D7_1360800) [17,47,52], and PfSPP [63]. Falcilysin has several functions, which is illustrated by at least two different EC classifications in EuPathDB EC.3.4.24.- (metalloendopeptidases) and EC.4.4.1.21 (S-ribosylhomocysteine lyase). This protease is present in the food vacuole, where it appears involved in hemoglobin catabolism [17,47], but additional isoforms generated by alternative splicing are also targeted to the *P.* *falciparum* apicoplast and mitochondrion, as described by Ralph et al. [55]. Regarding their different destinations, falcilysin may thus be present in three parasitic compartments: the digestive vacuole, the apicoplast by signal peptide cleavage, and the mitochondria by more complex splicing.

### M17 Peptidase family (leucyl aminopeptidase family)

The MF clan consists of aminopeptidases that need two cocatalytic metal ions (that could be Zn^2+^ and/or Mn^2+^) for activity. M17 is the only family represented in this clan; it is composed of leucine aminopeptidases (LAPs) [10]. These metalloexopeptidases catalyze the sequential removal of amino acids from the N-termini of proteins and peptidic substrates [56]. LAPs present two characteristic patterns: VGKG, corresponding to conserved amino acid regions, and NTDAEGRL, important for the active site [41].

In this *in silico* study, one LAP was found in the ToxoDB, referenced as TGME49_290670 and previously described by Jia et al. in 2010 [25]. This exopeptidase is localized in the cytoplasm of parasites and appears to be involved in free amino acid pool regulation. In 2015, Zheng et al. demonstrated that a *T. gondii* leucine aminopeptidase gene knockout influenced the growth of *T. gondii* without completely blocking parasite development, virulence or enzymatic activity [68]. We have found in the ToxoDB database that this LAP has an ortholog (NCLIV_042660) in the *N. caninum* genome, an expected situation considering the phylogenetic proximity of the two species.

Interestingly, we can also note that one M17 was identified in the *P. falciparum* genome as PF3D7_1446200, and this has been studied extensively [42,44,57,59]. This protease is expressed in all intra-erythrocytic stages and particularly at the trophozoite stage where protein synthesis increases [8,38]. It appears involved in the regulation of free amino acid spool [59]. Bestatin, a broad spectrum aminopeptidase inhibitor, prevents the growth of *P. falciparum* parasites *in vitro* and PNAP, a PfA-M17 specific inhibitor, blocks the malaria parasite development at ring stage, suggesting that this enzyme could play additional roles in the early erythrocytic development of the parasite [24]. Another apicomplexan LAP has also been characterized in *C. parvum* (CpLAP) that may also play an important role in free amino acid pool regulation [27]. Interestingly, TgA-M17 has a signal peptide contrary to PfA-M17 and TgA-M17 has wider substrate specificity than PfA-M17. While in the malaria parasite PfA-M17 is mainly described as a hemoglobinase, it could fulfill other roles [24]. Of note, TgA-M17 is currently related to glutathione metabolism (Kyoto Encyclopedia of Genes and Genomes KEGG metabolism) [26].

### M18 Peptidase family (Aminopeptidase I family)

M18 is part of the MH clan, and contains metallopeptidases that require two cocatalytic metal ions Zn^2+^. This family consists of aspartyl aminopeptidases (AAP), forming dodecameric complexes in humans, and exclusively cleaving aspartic or glutamic amino acids located at the N-termini of proteins and peptide chain [56]. As few AAP have been described in the literature, there is limited data on their enzymatic activities.

One *T. gondii* M18 has been identified in ToxoDB: TGME49_297970. This M18 peptidase, also called *Tg*AAP, is localized in the cytoplasm of the parasite and appears involved in parasite replication and growth [69].

In *P. falciparum*, Teuscher et al. [61] described *Pf*M18AAP octomers in the cytosol of parasites (synthesized during erythrocytic stages). *Pf*M18AAP (PF3D7_ 0932300) is exported and appears to act in synergy with other malarial aminopeptidases in order to achieve degradation of proteins such as hemoglobin. Antisense-mediated inhibition of PfM18AAP resulted in a lethal phenotype [61]. However, the involvement of this metallopeptidase in parasite survival remains controversial since Dalal and Klemba (2007) [14] were able to delete the gene without finding any deleterious effects, concluding that the protein function was not essential. This metallopeptidase is, however, considered a potential therapeutic target [58]. *Pf*M18AAP has also been shown to bind *in vitro* to human erythrocyte spectrin (spectrin binding region of 33 amino acids only present in *P. falciparum*), showing multiple enzymatic functions in the parasite and the erythrocytic host [36].

Recently, screening of inhibitors against malarial M1, M17 and M18 families have been tested using inhibitors present in the “Malaria Box”, allowing the identification of two potential inhibitors: MMV020750 and MMV666023 of PfA-M1 and PfA-M17, respectively [50].

### M20 Peptidase family (Glutamate carboxypeptidase, peptidase T, Xaa-His dipeptidase, carboxypeptidase Ss1 families)

Clan MH also presents the M20 family. This family encompasses glutamate carboxypeptidases and is characterized by the presence of two cocatalytic zinc metal ions, like in the M17 family [56]. Family M20 is currently divided into four separate sub-families: M20A, M20B, M20C and M20D.

One M20 peptidase was found in the *T. gondii* genome: TGME49_213520, but no experimental data concerning this or another *T. gondii* M20 peptidase has been described in the literature.

### M24 Peptidase family (Methionyl aminopeptidase 1 and aminopeptidase P families)

Clan MG contains exopeptidases that required two cocatalytic ions of cobalt and/or manganese, and contains only family M24. Peptidases belonging to this M24 peptidase family are also called methionine aminotransferase and cleave methionine residues at the N-terminal level. The M24 family has been divided into two sub-families: M24A (methionyl aminopeptidase) and M24B (X-pro aminopeptidase and X-pro dipeptidase). Most members of family M24 are cytosolic, and do not require proteolytic activation.

As of today, eight *T. gondii* methionine aminopeptidases have been found in ToxoDB with signature PFAM PF00557. Only one publication describes a *Toxoplasma* M24 peptidase (ToxoDB accession number: TGME49_261600) also called *Tg*APP (aminopeptidase P) and a recombinant form of this *Tg*APP has been expressed to evaluate its enzymatic parameters [67]. Deletion of the *Tg*APP gene in the parasite, through a CRISPR/Cas9 system, resulted in growth inhibition, thus indicating the importance of *Tg*APP as a potential therapeutic target [68].

Four M24 peptidases [62] have been published in *P.* *falciparum*, named *Pf*MetAP1a, *Pf*MetAP1b, *Pf*MetAP1c [11] and *Pf*MetAP2 [12], but only *Pf*MetAP1b was cloned, overexpressed, purified, and used to screen a compound library for inhibitors [11]. Interestingly, M24 peptidases have different localizations in *P. falciparum* parasites: *Pf*MetAP1a is present in mitochondria, *Pf*MetAP1b is present in cytosol and, *Pf*MetAP1c and *Pf*MetAP2 are in the apicoplast [16].

Other apicomplexa like *Cryptosporidium parvum*, *Eimeria tenella* or *Neospora caninum* encode for 5, 7 and 8 M24 metallopeptidases in their genomes, respectively (see also Table 3).

**Table 3:**
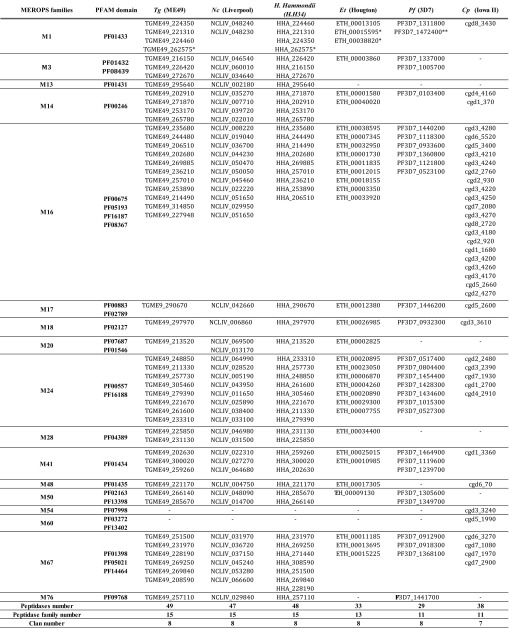
Comparative study of the metallopeptidase repertoires for *T. gondii* (*Tg*) strain ME49, *N. caninum* (*Nc*) strain Liverpool, *H. hammondii* strain HH34, *E. tenella* (Et) strain Houghton, *P. falciparum* (*Pf*) strain 3D7, and *C. parvum* (*Cp*) strain Iowa. Metallopeptidases are indicated by their EupathDB accession numbers and are classified into MEROPS families using PFAM domains and Blast similarity searches.

Besides the already mentioned and mostly studied malarial aminopeptidase PfA-M1 and PfA-M17 [28,53], these M24 malarial aminopeptidases also constitute very promising potential new targets for antimalarial drug development [62].

### M28 Peptidase family (aminopeptidase S, glutamate carboxypeptidase II, IAP aminopeptidase and aminopeptidase Ap1 families)

This family, included into clan MH, is composed of aminopeptidases and carboxypeptidases featuring two cocatalytic zinc ions [56].

Only two *T. gondii* M28 peptidases have been found in ToxoDB: TGME49_225850 and TGME49_231130. At the present time, no protein of this family has been experimentally described in *T.* *gondii* nor in other apicomplexa in the literature.

### M41 Peptidase family (FtsH endopeptidase family)

Clan MA(E), mentioned above, also includes family M41. Proteases of the M41 family are ATP-dependent metalloproteinases, also called FtsH peptidases [56]. These peptidases present the H**E**xxH motif and a third zinc ligand, which is a downstream aspartate. An ATPase domain follows the peptidase domain. In many bacteria, their activity increases as the temperature rises or during osmotic stress. These proteases thus play a role in protection against environmental stress [40].

In 2007, Karnataki et al. [29] identified a membrane-associated AAA (ATPases associated with diverse cellular activities) protease in *T. gondii* of the FtsH1 type (M41 peptidase family), corresponding to TGME49_259260. FtsH1 is an integral membrane protein which is targeted to the *T. gondii* apicoplast. From pulse-chase assays, the authors showed that two cleavages occurred within this protein sequence: a first one in the N-terminal part and a second one in the C-terminal part, allowing specific apicoplast targeting of this FtsH1 [29–30]. The authors suggested that the roles of FtsH1 in *T. gondii* could include protein surveillance, chaperone activity, and import [29]. Its function, however, has not yet been fully determined.

Three *T. gondii* M41 peptidases have been identified in ToxoDB: TGME49_202630, TGME49_200020 and TGME49_259260, among which only the latter has been described in the literature [29].

The *P. falciparum* genome encodes for three M41 peptidases. One of them (PF3D7_1239700) was identified as a AAA+/FtsH protease homolog (Pf FtsH1), exhibiting an ATP- and Zn^2+^-dependent protease activity and it has been localized in the *P. falciparum* mitochondria [60].

### M48 Peptidase family (Ste24 endopeptidase and HtpX peptidase families)

Also belonging to clan MA(E), the M48 family is divided into two sub-families: M48A (ste24 endopeptidase) and M48B (HtpX peptidase) [56].

Only one *T. gondii* metallopeptidase was identified in ToxoDB for this M48 peptidase family, TGME49_221170, but no protein of this family has been published to date. Other apicomplexa such as *C. parvum*, *E. tenella* or *N. caninum* also encode for one M48 metallopeptidase in their genomes, but *P. falciparum* does not seem to encode this enzyme.

### M50 Peptidase family (S2P peptidase and sporulation factor SpoIVFB families)

The M50 peptidase family consists of metalloendopeptidases with a single zinc in their active site, characteristic of clan MM. They form a distinct family of polytopic membrane metalloproteases containing 4 to 8 transmembrane domains. The M50 family presents a conserved 3 transmembrane domain core structure, containing the H**E**xxH motif within the first transmembrane domain of the core, and a second highly conserved motif called NxxPxxxxxxDG present in the third transmembrane domain; the three underlined amino-acids being the three zinc-ligands [56]. This M50 family has been divided into two sub-families: M50A (S2P protease) and M50B (sporulation factor SpoIVFB) [32–33].

As of today, no protein of this family has been described for *T.* *gondii* in the literature. Only two predicted proteases have been found in the genome of *T. gondii*: TGME49_266140 and TGME49_285670.

*Plasmodium* parasites encode in their genome two M50B-like proteases (PFAM13398): PF3D7_1305600 and PF3D7_1349700, according to Deu et al. (2017) [16], but lack the NxxPxxxxxxDG motif. In all invasive stages, the protein is in close proximity to the nucleus.

### M67 Peptidase family (Poh1 peptidase, JAMM-like protein and AMSH deubiquitinating peptidase families)

Clan MP contains a single family, M67 which presents divergent sequences divided into three sub-families: M67A (Poh1 peptidase component of the 26S proteasome), M67B (archean JAMM-like proteins), and M67C (AMSH deubiquitinating peptidase) [56]. The feature of their catalytic site motif is HxH, where the two underlined histidines provide zinc ligands together with an aspartate C-terminal to this motif; a glutamate N-terminal to this motif is a catalytic residue [56].

Six *T. gondii* peptidases have been identified in ToxoDB as belonging to this M67 family, none of which has been described in the literature to date.

However, two publications have described the proteasome of the malaria parasite, proposing enzymes involved in this pathway as promising drug targets for chemotherapeutic intervention as well as experimental evidence for metalloproteases in the proteasome complex [2,64]. In *T. gondii*, one publication described proteolytic activities in the proteasome, without indication of the presence of metalloprotease [51].

### M76 Peptidase family (Atp23 peptidase family)

These enzymes contain a H**E**xxH motif, in which E (bold) is a catalytic residue and the two H (underlined) are zinc-ion ligands (clan MA(E)), but the third zinc ligand has not yet been identified. The M76 peptidase family consists of endopeptidases whose functions are to achieve the synthesis of ATP from ADP and phosphate, a process occurring in mitochondria [56]. Only one *T. gondii* enzyme was found in ToxoDB: TGME49_257110, with a predicted localization within mitochondria. Yet, no member of this protease family has been described to date in the *T.* *gondii* literature.

### The enigma of M22 Peptidase family

During this study, we identified proteins ascribed to the “M22 peptidase family” in the Eupath database, including two members in the *T. gondii* genome, TGME49_274110 and TGME49_202310. While studying them, we however discovered that this family has been retracted from the MEROPS database, because there is a lack of experimental evidence to support peptidase activity as a general property of this family. The only evidence for any proteolytic activity in M22 was attributed to the O-sialoglycopeptidase from *Pasteurella haemolytica*. Homologs are almost universally distributed, but peptidase activity for members of this family has never been found. Structural studies have shown that members of “M22” have a very different fold to any known metallopeptidase (Rawlings, personal communication), and therefore they have been retracted from the MEROPS Database. Since the M22 domain signature continues to be present in the Eupath database and EMBL-EBI (Interpro service), we thought it was important to mention here that they are not members of the metallopeptidase superfamily, the focus of this current review.

## Conclusions

Metallopeptidases are of great importance in basic cell functions but also in specific cell functions. It is therefore necessary to inventory them for *T. gondii* as a way to better understand the biology of this parasite as well as the complexity of hosts and host-cell interactions. Also, with the aim of eventually undertaking a comparative study of apicomplexan genetic inheritance, it is worth mentioning that currently, *T. gondii* is the organisms that has the largest genome and encodes the highest number of genes, among all currently known apicomplexa.

At present, seven metalloproteases have been studied experimentally and described in *T. gondii*: an aminopeptidase N (family M1, aminopeptidase N) [5], two toxolysins (family M16, pitrilysin) [23,34], a leucine aminopeptidase (family M17, leucyl aminopeptidase) [25], an aspartyl aminopeptidase (family M18, aminopeptidase I) [69], a X-prolyl aminopeptidase (family M24, aminopeptidase P) [67], and a FtsH1 peptidase (family M41, FtsH peptidase) [29–30]. Out of these seven metalloproteases, only two have been shown to be involved in the invasion process of *T. gondii* within the host cell: toxolysins-1 and −4. The other metallopeptidases could be involved to various extents in a variety of metabolic pathways of *T. gondii*.

Overall, 49 metallopeptidases (7 published and 42 putative) containing various typical metallopeptidase signature motifs were identified in this study. Expression analysis of the corresponding 49 metallopeptidase genes in tachyzoite stages revealed the presence of transcripts for all of them, even if at low levels for some, such as M18 or M67 members for example. However, it would be interesting to adopt a quantitative PCR approach for each metallopeptidase, and thus to determine the expression levels of each.

Metalloproteases can be used to modify/degrade the host but also to activate some parasite proteins and they can be involved in egress, in invasion probably acting primarily as maturases, and in interactions with the host cell. *T. gondii* is also able to cross the basal membrane composed of laminin, Type III, IV and VII collagens, as well as glycosaminoglycans in order to diffuse in all organisms [4]. In addition, *T. gondii* must pass within the extracellular matrix composed of elastin and glycoproteins.

On the basis of the *in silico* study describing all putative and/or published metalloproteases in the *T. gondii* genome, we noted that some metalloprotease families were completely absent in the currently known apicomplexan peptidase families, and that some families were present only in one apicomplexan species: for example, peptidase family M54 and M60 are only present in *C. parvum*.

In conclusion, several families of metalloprotease are not represented in an identical manner depending on the parasite’s biology, physiology or host interaction, and could be potential therapeutic targets.

According to our comparative survey of metallopeptidases in 6 representative apicomplexan species (*T. gondii*, *N. caninum*, *H. hammondii*, *E. tenella*, *P. falciparum* and *C. parvum*), *T. gondii* together with *N. caninum* and *H. hammondii* contain the most numerous and diverse repertoire (49, 47, 48), followed by *C. parvum* (38), *E. tenella* (33) and then *P. falciparum* (29) (Table 3). This result is consistent with the recent observations by Woo et al., 2015 [65], indicating that the *T. gondii* genome would be currently the least reduced one − among all currently known apicomplexan genomes − compared to the genome that has been inferred for the apicomplexan common ancestor [39]. Besides having the largest number of metallopeptidases, *T. gondii,* *N. caninum* and *H. hammondii* also have the most diverse representation of metallopeptidases families (15), *P. falciparum* and *C. parvum* having the most reduced diversity (11 families), and *E. tenella* and intermediary status (13 families). The *C. parvum* repertoire is rather atypical with reduced diversity in terms of metallopeptidase families (11) but one of the largest sets of metallopeptidases (38), a situation that is due to remarkable expansion of the M16 family members in this species, the biological function of which will certainly deserve further investigations.

Interestingly, this comparative inventory reveals only two families that are evenly represented in the 6 representative species in terms of members: the M17 and M18 families, which each have a single member in the 6 species. For all the other metallopeptidase families there are many members (up to 20 for M16 in *C. parvum*) to none, possibly reflecting specific functions in the biology or host-parasite interactions of these species.

Thus, beyond its importance in providing novel putatively relevant targets for *T. gondii* chemotherapy, this inventory of *T. gondii* metallopeptidases provides the groundwork for functional investigations of their functions in parasite biology and host-parasite interactions of the diversity of apicomplexan parasites.

## Conflict of interest

The authors declare that they have no conflict of interest.

## Figures and Tables

**Appendix 1:**
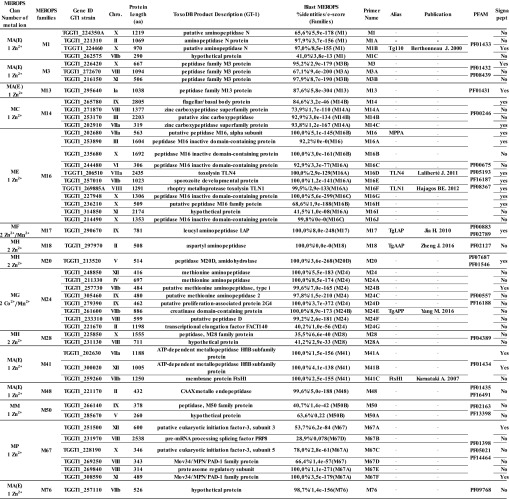
Metallopeptidase genes identified and classified in the *T. gondii* genome database (strain GT1, genotype I). We used Pfam motifs (http://pfam.xfam.org) in association with the MEROPS Database to screen the *T. gondii* database (http://toxodb.org/toxo/, Release 29). The motif organization of predicted peptidases was studied using the InterProScan Search (http://www.ebi.ac.uk/interpro/) and family assignment is based on MEROPS − the peptidase Database − classification (https://www.ebi.ac.uk/merops/).
